# Unravelling a Triad: A Rare Case of Takayasu Arteritis, Hashimoto’s Thyroiditis, and Psoriasis in a Young Female With Heart Failure

**DOI:** 10.7759/cureus.61153

**Published:** 2024-05-27

**Authors:** Mohak Jain, Minal Shastri, Nilay S Patel, Riya Dobariya

**Affiliations:** 1 Department of General Medicine, Sir Sayajirao General (SSG Hospital) Baroda Medical College, Vadodara, IND

**Keywords:** abdominal pain, computed tomography angiography, autoimmune disease, hashimoto thyroiditis, psoriasis, pulseless disease, congestive cardiac failure, takayasu arteritis

## Abstract

Takayasu arteritis (TA) is an autoimmune entity of unknown aetiology causing granulomatous thickening of large and medium-sized arteries. Common symptoms include claudication, headaches, dizziness, syncope, visual changes, and palpitations. Diverse cardiac manifestations, such as ischemic heart disease, significant aortic regurgitation, and pulmonary hypertension, are associated with TA, although they rarely manifest as congestive heart failure. Radio-imaging, including CT angiography and MR angiography, along with more invasive procedures such as conventional angiography, are often used for diagnosis. Treatment is done with corticosteroids, steroid-sparing agents, biologics, and revascularization procedures. Here, we have a case of a 17-year-old Indian female who presented to us with a complaint of abdominal pain. She was diagnosed with Hashimoto's thyroiditis a few years ago, along with a history of congestive heart failure. On general examination, blood pressure was asymmetrical in the upper limbs with the presence of bilateral carotid bruit. There was also the presence of extensive scaly lesions on the extensor surface of all four limbs, suggestive of psoriasis. Radio-imaging confirmed the diagnosis of TA. CT angiography also showed total occlusion of the celiac trunk and proximal left gastric artery, which was likely the cause of her symptoms. The patient received treatment with corticosteroids in conjunction with methotrexate, along with other supportive drugs. TA with congestive heart failure has been occasionally described in the literature, while the association of TA with psoriasis is much rarer. The simultaneous occurrence of various autoimmune diseases is common, but the triad of Hashimoto thyroiditis, psoriasis, and TA with an initial presentation of heart failure is unique. Due to the common co-occurrence of autoimmune conditions, early and thorough patient evaluation with comprehensive studies is imperative for optimal health outcomes.

## Introduction

Takayasu arteritis (TA), also known as pulseless disease, is an inflammatory autoimmune disease of unknown aetiology defined by granulomatous vasculitis affecting the aorta and its main branches and the pulmonary arteries. It is often characterized as an illness affecting women of childbearing age of Asian ethnicity. Common symptoms include claudication, headache, dizziness, syncope, dyspnea, visual changes, and palpitation, along with systemic symptoms like fever, night sweats, fatigue, malaise, and weight loss [1,2]. Diagnostic imaging like the computed tomography aortogram (CTA) is fundamental to diagnosis and disease monitoring [3]. Effective treatment includes immunosuppressive therapies, surgical revascularization, and management of comorbidities [4-6]. 

TA is associated with various cardiac manifestations like valvular insufficiencies, coronary artery disease, pulmonary hypertension, left ventricular (LV) hypertrophy, LV dysfunction, and thrombus formation [7]. The association of heart failure with TA is uncommon but has been reported [8,9]. It is often seen that various autoimmune diseases occur together. Previous studies have shown an association of TA with Hashimoto’s thyroiditis and TA with psoriatic arthritis [10-12]. However, the simultaneous occurrence of a trio of TA, Hashimoto’s thyroiditis, and psoriasis in congestive heart failure patients has not been documented in the literature to date.

Here, we put forth a case of a young female with hypothyroidism with a history of congestive heart failure provisionally diagnosed as dilated cardiomyopathy (DCM) of unknown aetiology. She presented to us with a complaint of abdominal pain and low-grade fever with new-onset skin lesions, which were eventually diagnosed as TA with psoriasis.

## Case presentation

A 17-year-old female, who had been a known case of Hashimoto's thyroiditis for two years, presented to us with chief complaints of abdominal pain for 15 days along with a low-grade fever. She experienced generalized abdominal pain, which increased sometime after eating food. She also complained of scaly skin lesions on both legs, forearms, and back. The patient denied any other abdominal complaints, lightheadedness, claudication, visual abnormalities, chest wall pain, or joint pain.

Upon eliciting her history, the patient revealed a history of heart failure two years prior. In 2022, she experienced breathlessness and received a provisional diagnosis of DCM. A detailed workup to identify the cause of DCM was not done due to the financial constraints of the patient. The two-dimensional echocardiography (2D-echo) done then showed moderate left ventricle (LV) systolic dysfunction; the left ventricle ejection fraction (LVEF) was 35-40% with moderate global hypokinesia. The left atrium (LA) and LV were dilated with the presence of moderate to severe mitral regurgitation (MR) and moderate pulmonary artery hypertension (PAH) with a right ventricle systolic pressure (RVSP) of 55 mmHg. The patient was also diagnosed with hypothyroidism at that time. Anti-TPO was elevated to 261.50 IU/mL, which was suggestive of Hashimoto’s thyroiditis. The patient had a positive family history of autoimmune conditions; her maternal uncle had a history of transverse myelitis with positive anti-MOG antibodies. Medical therapy was started (which included beta-blockers, diuretics, and ACE inhibitors), and the patient improved thereafter. She was also started on thyroxine. Her thyroid profile was also, consequently, normal with medical therapy.

**Figure 1:**
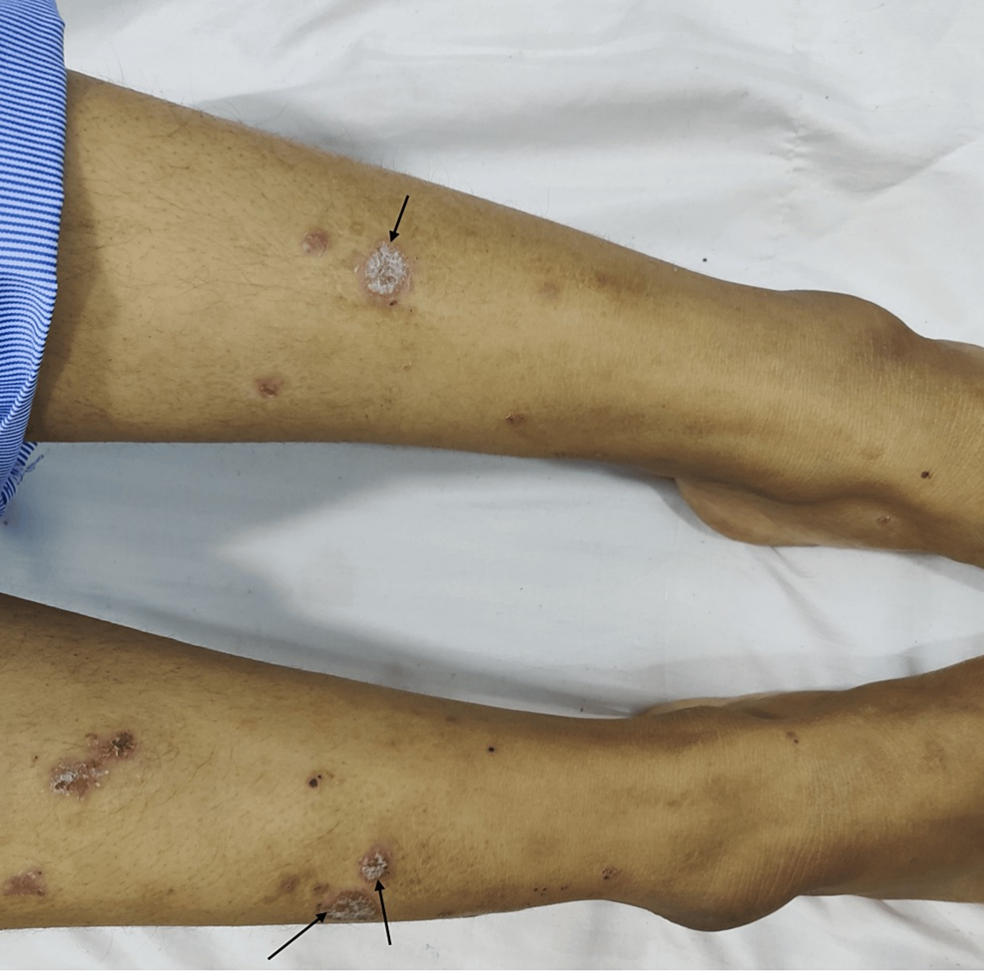
Variably sized, scaly, erythematous plaques (arrows) present over the extensor surfaces of lower limbs suggestive of Psoriasis.

The physical examination at the time of presentation in our hospital revealed a slightly raised temperature and a weak right radial artery pulse, with blood pressure measuring 110/70 mmHg in the left arm and 70 mmHg systolic blood pressure in the right arm. The blood pressure in both lower limbs was 240/130 mmHg. Examination revealed a carotid thrill on palpation along with a bruit on auscultation. Skin examination revealed extensive lesions, including slightly scaled, variably sized, coalescent erythema or plaques on all extremities and the back (Figure 1). The candle wax sign and Auspitz's sign were positive, which prompted the diagnosis of psoriasis vulgaris.

Routine investigation (Table 1) showed severe microcytic hypochromic anaemia with decreased haemoglobin (Hb), hematocrit (HCT), red cell indices, and increased red cell distribution width (RDW), likely suggestive of iron deficiency. The total white cell count and platelet count were normal. The elevated levels of C-reactive protein (CRP) and erythrocyte sedimentation rate (ESR) were noted. Ferritin was within the reference range, with normal SGPT, S. urea, and S. creatinine. Urinalysis was normal. The thyroid profile showed low T3 and TSH with normal T4 on regular medication, indicative of sick euthyroid syndrome.

**Table 1:** Routine investigations. ESR: erythrocyte sedimentation rate, SGPT: serum glutamic pyruvic transaminase; pg: picogram; IU: international units; fL: femtoliter.

Investigation	Value	Reference range
Haemoglobin (g/dL)	6.6	11.9–14.8 (g/dL)
Hematocrit (%)	25.4	35–43 (%)
Mean corpuscular volume (fL)	60.1	82.5–98 (fL)
Mean corpuscular haemoglobin (pg)	15.6	28.4–30.7 (pg)
Mean corpuscular haemoglobin concentration (g/dL)	25.9	33.9–35.4 (g/dL)
Red cell distribution Width (fL)	21.1	11.4–13.5 (fL)
Total White Cell Count (per cumm)	9860	3800–10,400 (per cumm)
Platelet Count (per cumm)	318000	150,000–410,000 (per cumm)
C-reactive protein (CRP) (mg/L)	134.23	0–10 (mg/dL)
ESR (mm/1 hr)	34	0–15 (mm/1 hr)
Ferritin (ng/mL)	47.81	24–336 (ng/mL)
Serum urea (mg/dL)	14	14–40 (mg/dL)
Serum creatinine (mg/dL)	0.8	0.52–1.04 (mg/dL)
SGPT (IU/L)	10	0–35 (IU/L)

Electrocardiography (ECG) showed high-amplitude QRS complexes in precordial leads, with the Katz-Wachtel phenomenon suggesting biventricular hypertrophy. The chest radiogram showed normal lung fields without any visible abnormalities.

**Figure 2:**
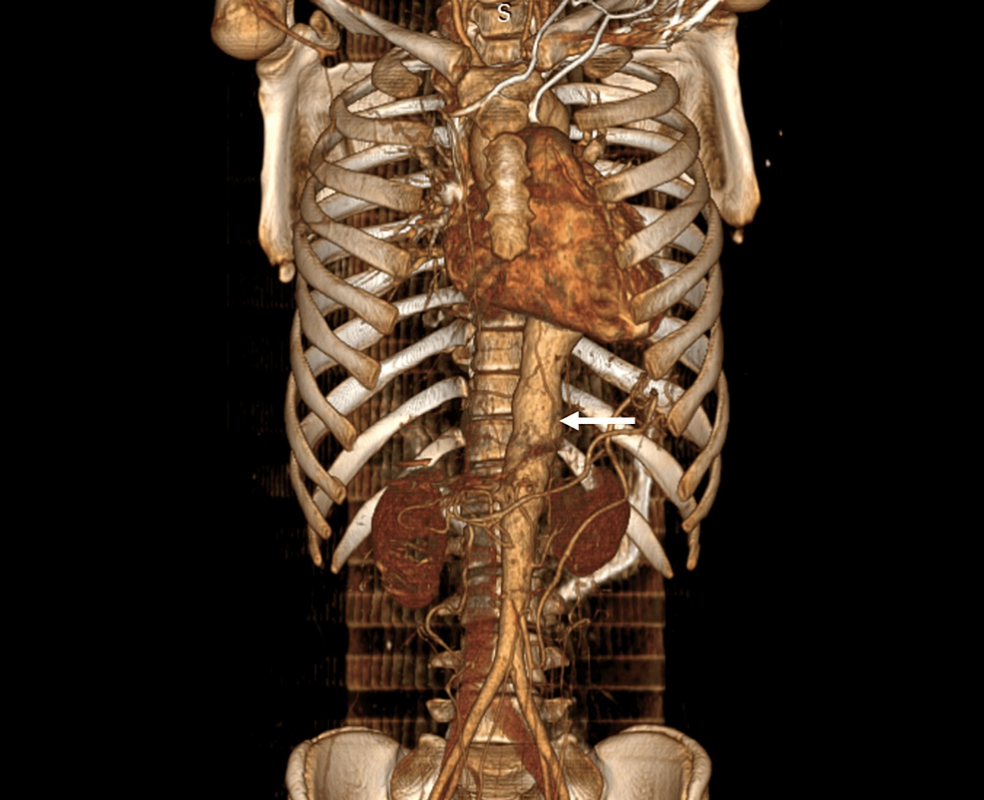
Three-dimensional render of CT aortogram showing irregularly dilated thoracic aorta (white arrow) with non-visualization of several of its branches.

Radiologic investigations like ultrasonography (USG) and CTA were suggestive of TA, and 2D-Echo was suggestive of moderate aortic regurgitation (AR) with grade 2 diastolic dysfunction (Table 2, Figure 2).

**Table 2:** Radiological investigations. LV: left ventricle, CT: computed tomography, USG: ultrasonography. A CT aortogram showed complete occlusion as well as narrowing of many abdominal and thoracic arteries, which led to symptoms of abdominal pain. It also caused hypertension (due to renal artery stenosis), asymmetrical blood pressure readings, and carotid bruit. It is also the likely reason for resultant aortic regurgitation and an antecedent history of congestive heart failure.

Investigation	Comments
USG of the abdomen and pelvis	Abnormal dilatation of aorta
CT aortogram	Total occlusion: celiac trunk, splenic artery, common hepatic artery, proximal left gastric artery, bilateral subclavian artery distal to vertebral artery origin.
	Significant narrowing: superior mesenteric artery, upper pole branch of the right renal artery, left proximal external carotid artery with moderate dilatation of the carotid bulb.
	Moderate narrowing: left common carotid artery.
	Mild narrowing: a cervical segment of the left internal carotid artery, right proximal common carotid artery with moderate dilatation of the distal segment, bilateral vertebral arteries near the origin, brachiocephalic trunk, and proximal descending thoracic aorta.
	Patent artery: left accessory renal artery.
Two-dimensional echocardiography (2D-ECHO)	Normal LV with fair LV function, with no resting wall motion abnormality at rest, left ventricle ejection fraction was 55%, there was grade ll diastolic dysfunction with trivial mitral regurgitation, mild- moderate aortic regurgitation.

Prior to the diagnosis of TA, the patient was taking carvedilol (3.125 mg) once a day and thyroxine (75 μg). She was then started on prednisone (40 mg/day) with gradual tapering along with methotrexate. Local therapy, including emollients and supportive drugs, was given for psoriasis. Iron replenishment was also done. After five days of hospitalization, the patient’s general condition and blood parameters improved, and the patient was discharged with advice to follow up monthly.

On follow-up, the patient had significant improvement in symptoms, with a decrease in abdominal pain. Peripheral pulsations also improved, with a blood pressure of 100/70 mmHg in both upper limbs, although it remained high in both lower limbs (180/100 mmHg). Inflammatory markers also returned to near normal (ESR 18 mm/hr). Angioplasty was then planned for renal artery stenosis, and conservative medical management was continued with follow-up radio-imaging to assess disease remission in the remainder of the vessels.

## Discussion

TA is a chronic inflammatory disease of unknown aetiology that primarily involves the aorta and its major branches. It is usually present in Asian females less than 40 years of age [1]. Our patient presented at 17 years of age with Hashimoto’s thyroiditis and psoriasis. TA usually presents with fever, night sweats, arthritis, ocular disturbances, neurological symptoms, claudication, blood pressure discrepancies, bruits, and chest pain [1]. Abdominal pain as the chief complaint is rare because of the less frequent incidence of involvement of the celiac trunk in TA [13]. However, a predisposing symptom in our patient was abdominal pain because of the total occlusion of the celiac trunk noted on the CTA. On general examination, a weak pulse, blood pressure discrepancies, carotid thrill, and bruit were noted, along with significant skin lesions of psoriasis. 

Laboratory evaluation in TA reveals normochromic, normocytic anaemia, leukocytosis, thrombocytosis, elevated ESR, CRP, and raised ferritin [14]. However, in our case, microcytic hypochromic anaemia, along with apparently normal ferritin and a high ESR and CRP, were likely due to a concomitant iron deficient status along with a generalized inflammatory state.

Imaging is fundamental to the diagnosis and monitoring of TA. Imaging evaluation includes a non-invasive approach via USG, colour doppler ultrasound (CDUS), CTA, magnetic resonance angiography (MRA), 18F-fluorodeoxyglucose-labelled positron emission tomography (18 FDG-PET), and an invasive approach like catheter-directed injection of intravascular dye, which provides useful information on vascular luminal dimensions [1,3,15]. In our case, the patient had undergone abdominal USG for abdominal pain and was found to have abnormal aortic dilatation, following which a CTA was done for further evaluation and confirmation of TA, which showed total occlusion of the celiac trunk and its branches, along with bilateral subclavian artery distal to vertebral artery origin. Although granulomatous inflammation, elasophagia, adventitial, and medial fibrosis on histopathology are confirmatory, they are largely replaced by imaging modalities [16].

Although TA is rarely associated with cardiomyopathy [12], our patient experienced congestive heart failure. Initially, it was misdiagnosed as DCM two years ago, but it responded well to medical therapy. The follow-up 2D-Echo showed normal LV size and function (LVEF = 55%) with grade 2 diastolic dysfunction with trivial MR and mild-moderate AR, which was likely due to TA. The ECG evaluation was also suggestive of biventricular hypertrophy. TA is also rarely associated with psoriasis [12], which, as seen in our case, warrants a need for detailed genetic evaluation to establish a causal link.

Treatment options in TA include higher-dose glucocorticoids (GC), non-GC non-biologic therapies such as methotrexate, mycophenolate mofetil, and azathioprine, biologics such as TNF inhibitors (including infliximab, etanercept, and adalimumab), and tocilizumab [4,5]. Vascular reconstructive surgery is needed for up to 70% of patients with TA [6]. In our case, the patient was treated with prednisolone 40 mg/day for a month with gradual tapering doses and the addition of methotrexate. Iron replenishment was also done for comorbid anemia. Apart from methotrexate and oral glucocorticoids, psoriasis management also involves the use of emollients and supportive drugs.

## Conclusions

Takayasu’s arteritis is a rare type of autoimmune inflammatory vasculitis, commonly occurring in females of Asian ethnicity. It is common to find various autoimmune diseases coexisting. However, it is rare to find a combination of Hashimoto's thyroiditis, psoriasis vulgaris, and TA together, as depicted in our case. While there have been occasional cases of psoriasis with TA, detailed genetic associations remain to be elucidated. Further research is needed in this regard. Dermatologists need to be aware of this unique association between psoriasis and TA. Although identifying the aetiology of heart failure can be challenging, this case study highlights the need for a thorough clinical examination to identify the aetiology. Prompt diagnosis of autoimmune disorders and their cardiac manifestations in a timely manner is crucial to avoid potentially harmful consequences.
